# Axillary Lymph Node Dissection for Breast Cancer: Efficacy and Complication in Developing Countries

**DOI:** 10.1200/JGO.18.00080

**Published:** 2018-10-03

**Authors:** Mohaned O. Abass, Mohamed D.A. Gismalla, Ahmed A. Alsheikh, Moawia M.A. Elhassan

**Affiliations:** **Mohaned O. Abass**, Shendi University, Shendi, River Nile State; and **Mohamed D.A. Gismalla**, **Ahmed A. Alsheikh**, and **Moawia M.A. Elhassan**, University of Gezira, Wad Medani, Gezira State, Sudan.

## Abstract

**Purpose:**

Axillary lymph node dissection (ALND) frequently is performed as part of the surgical management of breast cancer as a therapeutic and prognostic index, but increasingly has been perceived as associated with significant complications. Data on efficacy and complications of ALND in Sudan are lacking. The aim of this study was to assess the efficacy and complications of ALND in patients with breast cancer treated with mastectomy and breast-conserving surgery.

**Methods:**

We performed a prospective, hospital-based study in women with invasive breast cancer who underwent modified radical mastectomy or breast-conserving surgery with ALND between September 2014 and August 2015. The efficacy of ALND was defined as retrieval of ≥ 10 lymph nodes. Complications of ALND were assessed objectively and subjectively and defined as either present or absent.

**Results:**

Of 96 patients with breast cancer included in the study, 40 (42%) developed postaxillary clearance complications. The median follow-up time was 18 months (range, 12 to 24 months). Numbness was reported by 21.9% of patients. Seroma was noted in 15.6% and lymphedema in 9.4%. Approximately 9% reported episodes of infection or inflammation at the surgical site. None of the studied factors were found to affect the incidence of complications significantly. Ten or more lymph nodes were retrieved in 81.3% of patients, and nodal metastasis was found in 62.5%.

**Conclusion:**

This study shows that the prevalence of undesirable adverse effects after ALND is 42%, with paresthesia and seroma being the most prevalent. The lymphedema prevalence was low in relation to other evaluated symptoms.

## INTRODUCTION

Breast cancer is the most frequently diagnosed cancer in women worldwide and is second only to lung cancer as a leading cause of cancer-related death. In developing countries, breast cancer incidence and mortality have been rising.^1^ In Sudan, breast cancer continues to be the most common cancer among women and constitutes 20% of all cancer diagnoses registered at the Gezira National Cancer Institute (NCI). The incidence of breast cancer in Khartoum State, Sudan, was found to be 25.1 per 100,000.^2,3^ Breast cancer in Sudanese women is characterized by younger age at onset and advanced stage at diagnosis, with a high incidence of locoregional recurrence.^4-6^

Although axillary lymph node dissection (ALND) has largely been replaced by sentinel lymph node biopsy (SLNB) for patients with cN0 breast cancer,^7^ this advanced technique is presently not available in Sudan.^5^ Therefore, ALND is recommended as part of the primary surgical management of patients with invasive breast cancer in our setting according to Sudan national guidelines for breast cancer management.^8^

ALND plays an essential role in the surgical management of breast cancer. The information obtained from pathologic examination of the removed lymph nodes helps to determine the pathologic staging of the disease and is an integral part of the treatment of breast cancer.^9^ ALND is beneficial for patients with breast cancer because it controls regional nodal disease and may improve overall survival.^10^ Complications after ALND are well recognized and include wound infection, lymphedema of the arm, lymphangitis, arm numbness, and limitation of arm movement.^11,12^

In Sudan, where level 1 and 2 ALND is required for almost all patients with invasive breast cancer, the efficacy and incidence of the complications of ALND are unknown. Thus, we provide baseline information about the efficacy and complications of ALND in Sudanese patients who undergo modified radical mastectomy (MRM) and breast-conserving surgery (BCS).

## METHODS

### Setting

Surgical operations were performed at Wad Medani Teaching Hospital located in the city of Wad Medani, the capital of Gezira State, Sudan, which serves all of Gezira and nearby states. Histopathologic and cytopathologic studies are provided by the University of Gezira Medical Laboratory. The Gezira NCI is the only cancer treatment facility in the state. The cancer treatment modalities available at NCI include radiotherapy (cobalt-60 machines), chemotherapy, and palliative care. A complete clinical work-up (eg, ultrasound, x-rays, blood tests, bone marrow examination, tumor marker analysis, nuclear imaging) are available at NCI.

### Study Design

We performed a prospective, descriptive, cross-sectional hospital-based study to evaluate the efficacy and characterize the incidence of ALND complications in patients who underwent MRM and BCS at Wad Medani Teaching Hospital between September 2014 and August 2015.

### Inclusion Criteria

Women with histologically confirmed invasive breast carcinoma without distant metastases (M0) at diagnosis were included in this study. All patients underwent level 1 and 2 ALND associated with mastectomy or BCS.

### Exclusion Criteria

Patients with other malignancies, fractures, or previous surgery in the upper limb ipsilateral to the ALND were excluded from this study.

### Data Collection

All patients with breast cancer are evaluated in our breast multidisciplinary team meeting at NCI. Patients who fulfilled the inclusion criteria for the study were consented to be included in the study. We used a predesigned questionnaire to collect data from patient folders, histopathology reports, and patients during preoperative clinical assessment; after surgery during hospital admission; and at 2-, 6-, 12-, and 18-month follow-up. The data collected were patient and tumor characteristics; type of surgery; number of lymph nodes retrieved by pathologists; number of lymph nodes positive for metastasis; and complications of ALND, which included lymphedema, seroma, paresthesia, pain, infection, shoulder weakness, and restriction of arm movement. The evaluation results were defined as either present or absent.

### Outcomes

The efficacy of ALND was defined as the retrieval of ≥ 10 lymph nodes. Lymphedema was defined as a difference > 2 cm in the upper arm circumference between the arm ipsilateral to the ALND and the nonsurgical arm. Seroma was defined as accumulation of fluid that was either aspirated or treated conservatively in the axillary space after the discontinuance of the drain. Restriction of arm movement was defined as any degree of restriction in abduction of the arm ipsilateral to ALND. Postoperative infection referred to patients who were prescribed antibiotics with the intention to treat infection. Pain and paresthesia assessment relied on patient-reported symptoms.

### Assessment Tools

The following objective and subjective tools were used to assess complications of ALND. 

#### Objective tools.

Circumference of the upper arm ipsilateral to the ALND was measured by the clinician and compared with the contralateral nonsurgical arm. Range of motion was assessed by the surgeon as active ranging at the shoulder joint, which was scored as equal to or decreased relative to the nonoperated side. Total drain outputs were recorded daily for all patients. The drains were removed when the daily drainage volume was < 100 mL. Accumulation of fluid in the axillary space that was either aspirated or treated conservatively after the discontinuance of a drain was considered as seroma.

#### Subjective tools.

Pain level was evaluated by directly asking the patient about the presence or absence of arm pain. Paresthesia assessment relied on patient-reported symptoms.

### Data Analysis

Data were entered and analyzed using SPSS version 21 statistical software (IBM Corporation, Chicago, IL). Both descriptive and inferential statistics (independent *t* test) were used to present results. Numerical data were expressed as means and standard deviations (SDs). Results were tabulated and presented as frequencies and percentages, as appropriate. For each test, *P* < .05 was considered statistically significant (95% CI).

## RESULTS

Ninety-six patients with breast cancer were included in this study. The median follow-up time was 18 months (range, 12 to 24 months). Median age was 45 years (range, 25 to 85 years). Patient and treatment characteristics are listed in Table 1. The mean pathologic tumor size was 4.45 cm (SD, 2.10 cm). The distribution of stage I, II, and III breast cancer was 3%, 49%, and 48%, respectively.

**Table 1:**
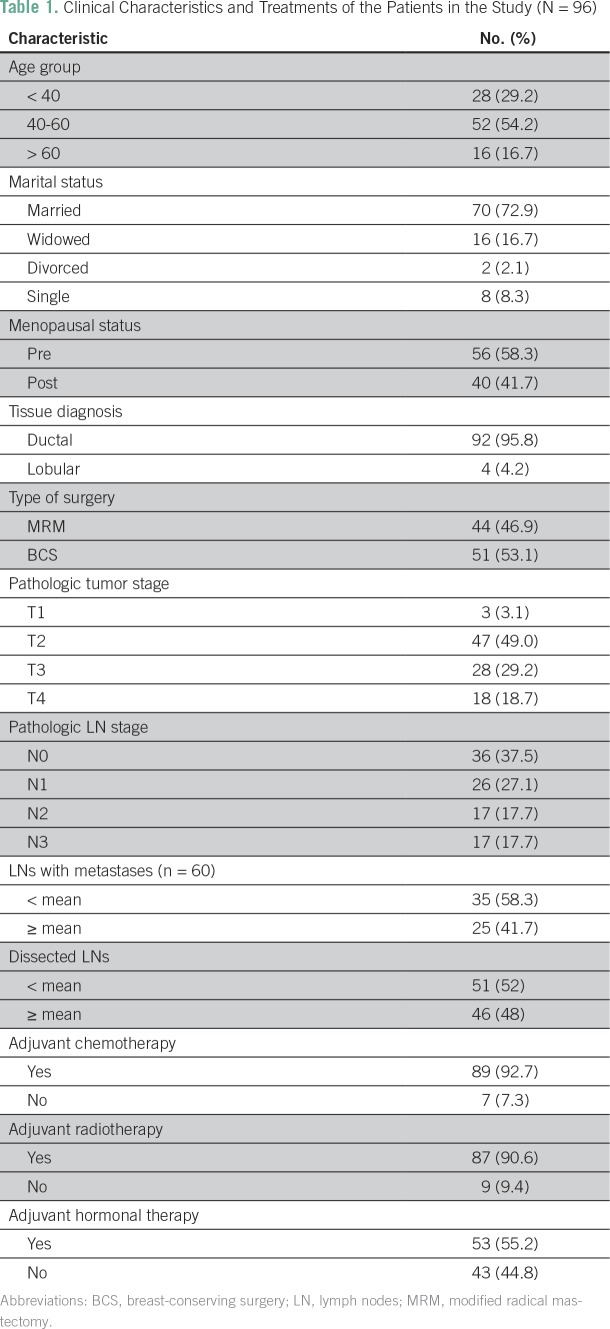
Clinical Characteristics and Treatments of the Patients in the Study (N = 96)

Forty-five patients (46.9%) underwent MRM, and 51 (53.1%) underwent BCS and axillary dissection. Axillary lymph nodes were palpable in 45% of patients. Fifteen patients (15.6%) underwent surgery after receiving neoadjuvant chemotherapy (NACT). Fifty-five procedures (57.3%) were performed by surgeons, whereas 41 (42.7%) were done by senior registrars. The mean postoperative hospital stay was 4.25 days (range, 2 to 9 days).

All patients underwent level 1 and level 2 ALND. The median and mean number of lymph nodes retrieved were 14 (range, two to 31) and 15 (SD, 6.0), respectively. We found that in 81.3% of the study population, ≥ 10 lymph nodes were retrieved. Of the 15 patients who received NACT, ≥ 10 lymph nodes were retrieved from 12 (80%), whereas of 81 patients who did not receive NACT, ≥ 10 lymph nodes were retrieved from 66 (81%). The mean number of lymph nodes with metastasis was seven (SD, 5.9), and 60 patients (62.5%) had at least one positive lymph node.

**Fig 1:**
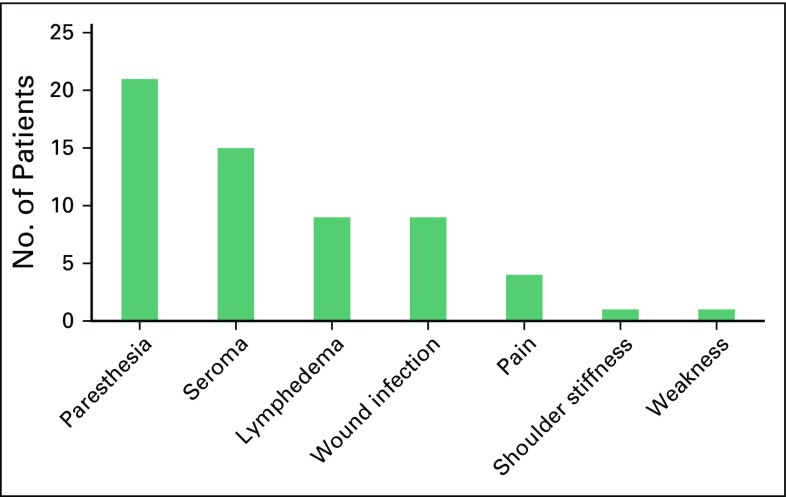
Prevalence of axillary lymph node dissection complications in patients with breast cancer treated with modified radical mastectomy or breast-conserving surgery.

Of all patients in this study, 41 (42.7%) developed postoperative complications. Paresthesia was the most frequent complication (21.9%) followed by seroma (15.6%; Fig 1). No statistically significant differences were found between the different variables and complications (Table 2). The pattern of complications according to type of surgery, surgeon experience, and status of axillary lymph nodes are listed in Table 3. All wound infections were local surgical infections and responded well to daily dressing, and secondary suture or hospital admissions for intravenous antibiotics were not needed.

**Table 2:**
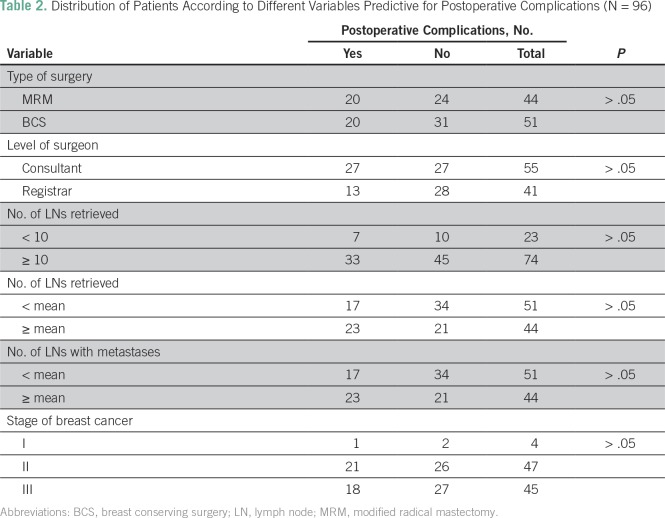
Distribution of Patients According to Different Variables Predictive for Postoperative Complications (N = 96)

**Table 3:**
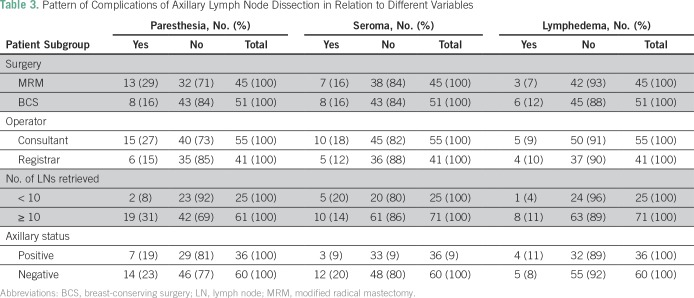
Pattern of Complications of Axillary Lymph Node Dissection in Relation to Different Variables

Fifteen patients (15.3%) received NACT. Among them, none had pN0, one had pN1, two had pN2, and 12 had pN3 disease. Nine (60%) of the 15 patients who received NACT developed complications (four paresthesia, two seroma, two lymphedema, and one infection).

## DISCUSSION

To our knowledge, this study is the first of its kind in Sudan to evaluate the efficacy and complications of ALND in patients who undergo MRM or BCS. Treatment of axilla as part of the treatment of patients with breast cancer also has changed over time, and various options are available, including axillary dissection, axillary clearance, axillary dissection with regional lymph node radiation, regional radiation alone, axillary sampling, endoscopic axillary clearance, SLNB, and observation. Complications of ALND are increased by the extent of the dissection.^13,14^

In currently accepted guidelines, the removal of ≥ 10 axillary nodes represents the international gold standard for systematic axillary staging.^15^ In this study, ≥ 10 lymph nodes were removed from > 80% of patients. Surgical procedures were performed by consultants as well as registrars at a nearly similar distribution. We found no significant differences between consultants and residents with regard to number of lymph nodes removed during ALND.

Of the patients in this study, 40.4% developed complications after ALND. Paresthesia and seroma were the most prevalent adverse effects. The lymphedema prevalence was low relative to other evaluated symptoms. In this study, no statistically significant differences were found between the different variables and complications. The literature reports that the risk of complications correlates positively with the radical nature of ALND.^14^

ALND was replaced in clinical practice by SLNB in patients without axillary lymph node involvement (N0) and in some with N1 disease.^16^ In the current study, approximately one third of patients with negative lymph nodes developed postoperative complications. The rate of complications in such patients can be minimized by using SLNB. In limited-resource settings, SLNB actually can be used at a low cost when the sentinel node mapping is restricted to the use of blue dye without radiotracer. In Sudan, although nuclear medicine services are available, SLNB as a technique for the evaluation of axillary lymph nodes in patients with breast cancer is lacking.

We found that approximately one half of the patients studied had positive nodes and on the basis of current evidence, would be recommended for axillary dissection. Therefore, strategies to prevent possible complications of ALND should be followed to reduce the incidence and severity of these complications. Categories of preventing lymphedema include avoidance of trauma, prevention of infection, and use and exercise of the limb. Closed suction drainage after ALND is advantageous in decreasing the incidence and degree of seroma formation. Modest mobility of the arm must be encouraged to minimize stiffness and frozen shoulder. Surgeons should preserve the intercostobrachial nerve whenever possible to minimize the risk of numbness or paresthesia of the upper medial arm and/or axilla.

Lymphedema, the most serious and difficult-to-treat complication, occurred in nine patients (9.4%). This finding is nearly equal to the rate of lymphedema reported in the American College of Surgeons Oncology Group trial Z0011^17^ and less than that mentioned in other studies.^18,19^ The variation in incidence of lymphedema could be due to great variability in procedures, radiation treatments, objective assessment criteria, and duration of follow-up. Incidence of lymphedema seemed to increase with time up to 2 years after diagnosis or surgery, after which incidence seemed to decrease.^20^

Paresthesia was the most frequent complication in this study and was found in 20% of patients compared with 35% to 68% reported in other studies.^21,22^ Paresthesia is related to the intercostobrachial nerve section that crosses the axilla and is transected during ALND. The low incidence found in this study could be due to difficulty in assessing paresthesia after axillary dissection in the immediate postoperative period. Moreover, paresthesia does not limit quality of life in most patients, and many patients will not complain about it.

Despite the use of postoperative closed suction drainage to minimize prolonged seroma formation, we found that 15% of patients developed seroma after the discontinuance of the drain. During the study period, we found a very low incidence of wound infection. No patients were hospitalized for the treatment of arm infection with intravenous antibiotics or required secondary suture. Although NACT is associated with an increased risk of postoperative infection, only one of the 15 patients who received NACT developed wound infections. In Sudan, locally advanced breast cancer is a common clinical scenario. In a selected group of patients, the use of NACT allows for significant downstaging of the primary tumor and lymph node metastases, which permits subsequent BCS or mastectomy with a greater expectation of long‐term success.

The incidence of pain and range-of-motion restriction varies widely in the literature. We identified a very low incidence of arm movement limitation and pain. Our findings are low compared with a previous study conducted by Warmuth et al,^21^ who reported an 8% and 30% incidence of arm movement limitation and pain, respectively, at 5 years after ALND. The low incidence reported in the current study could be due to a short follow-up period after ALND.

Approximately two thirds of the current study population had metastases in axillary lymph nodes. Previous studies from Sudan reported that the majority of patients with breast cancer were diagnosed at advanced stages.^4,5,23^ Ahmed^24^ highlighted the possible reasons for late presentation of Sudanese patients with breast cancer and concluded that lack of education, a dependency on traditional medicine, and financial aspects of undergoing testing and treatment are the most important factors that play a role in prolonging the patient’s decision to seek medical treatment. In our setting, most patients with breast cancer are not candidates for SLNB because of advanced stage at presentation. Application of SLNB in countries with limited resources like ours warrants widespread support from relevant stakeholders, including medical personnel and policymakers.^25^

This study had several limitations. First, its cross-sectional design could lead to recall bias, and the small sample size included patients from only one tertiary hospital in Sudan. Thus, complications seen in other regions of Sudan likely differ from those reported here. Second, we did not use standardized grading of the complication severity, and the assessment results were defined as either present or absent. Third, we did not report which complications resolved partially or completely over the study period. Fourth, the relation among various risk factors such as body mass index, immunosuppression, and medical history of type II diabetes mellitus, which are known to increase the incidence of complications after ALND, were not evaluated. Finally, surgeons did not report whether the intercostobrachial nerve was preserved; in some difficult dissections, the nerve may have been injured.

In conclusion, this study provides baseline information about the complications of ALND in Sudanese patients with breast cancer treated with mastectomy and BCS. The incidence of ALND adverse effects in this study was 43%. Paresthesia and seroma were the most frequent adverse effects. Lymphedema prevalence was low in relation to other evaluated symptoms. A multi-institutional study that uses objective measures and standardized grading of complication severity over a long follow-up period is needed to determine better the burden and pattern of complications and their evolution over time.
